# Assessing *Wolbachia*-mediated sterility for dengue control: emulation of a cluster-randomized target trial in Singapore

**DOI:** 10.1093/jtm/taae103

**Published:** 2024-08-06

**Authors:** Jue Tao Lim, Diyar Mailepessov, Chee-Seng Chong, Borame Dickens, Yee Ling Lai, Youming Ng, Lu Deng, Caleb Lee, Li Yun Tan, Grace Chain, Soon Hoe Ho, Chia-Chen Chang, Pei Ma, Somya Bansal, Vernon Lee, Shuzhen Sim, Cheong Huat Tan, Lee Ching Ng

**Affiliations:** Lee Kong Chian School of Medicine, Nanyang Technological University, 11 Mandalay Rd, 308232, Singapore; Environmental Health Institute, National Environment Agency, 11 Biopolis Wy, 138667, Singapore; Environmental Health Institute, National Environment Agency, 11 Biopolis Wy, 138667, Singapore; Environmental Health Institute, National Environment Agency, 11 Biopolis Wy, 138667, Singapore; Saw Swee Hock School of Public Health, National University of Singapore and National University Health System, 12 Science Drive 2, #10-01, 117549, Singapore; Environmental Health Institute, National Environment Agency, 11 Biopolis Wy, 138667, Singapore; Environmental Health Institute, National Environment Agency, 11 Biopolis Wy, 138667, Singapore; Environmental Health Institute, National Environment Agency, 11 Biopolis Wy, 138667, Singapore; Environmental Health Institute, National Environment Agency, 11 Biopolis Wy, 138667, Singapore; Environmental Health Institute, National Environment Agency, 11 Biopolis Wy, 138667, Singapore; Environmental Health Institute, National Environment Agency, 11 Biopolis Wy, 138667, Singapore; Environmental Health Institute, National Environment Agency, 11 Biopolis Wy, 138667, Singapore; Environmental Health Institute, National Environment Agency, 11 Biopolis Wy, 138667, Singapore; Saw Swee Hock School of Public Health, National University of Singapore and National University Health System, 12 Science Drive 2, #10-01, 117549, Singapore; Saw Swee Hock School of Public Health, National University of Singapore and National University Health System, 12 Science Drive 2, #10-01, 117549, Singapore; Ministry of Health, 16 College Road College of Medicine Building, 169854, Singapore; Environmental Health Institute, National Environment Agency, 11 Biopolis Wy, 138667, Singapore; Environmental Health Institute, National Environment Agency, 11 Biopolis Wy, 138667, Singapore; Environmental Health Institute, National Environment Agency, 11 Biopolis Wy, 138667, Singapore; Saw Swee Hock School of Public Health, National University of Singapore and National University Health System, 12 Science Drive 2, #10-01, 117549, Singapore; School of Biological Sciences, Nanyang Technological University, 60 Nanyang Dr, 637551, Singapore

**Keywords:** Efficacy, Wolbachia, dengue, vector control, IIT, SIT, IIT-SIT

## Abstract

**Background:**

Matings between male *Aedes aegypti* mosquitoes infected with *w*AlbB strain of *Wolbachia* and wildtype females yield non-viable eggs. We evaluated the efficacy of releasing *w*AlbB-infected *Ae. aegypti* male mosquitoes to suppress dengue.

**Methods:**

We specified the protocol of a two-arm cluster-randomized test-negative controlled trial (cRCT) and emulated it using a nationally representative test-negative/positive database of individuals reporting for febrile illness to any public hospital, general practitioner or polyclinic. We retrospectively built a cohort of individuals who reside in *Wolbachia* locations vs a comparator control group who do not reside in *Wolbachia* locations, using a nationally representative database of all individuals whom report for febrile illness and were tested for dengue at the Environmental Health Institute/hospital laboratories/commercial diagnostic laboratories, through general practitioner clinic, polyclinic or public/private hospital from epidemiological week (EW) 1 2019 to EW26 2022. We emulated a constrained randomization protocol used in cRCTs to balance dengue risk between intervention and control arms in the pre-intervention period. We used the inverse probability weighting approach to further balance the intervention and control groups using a battery of algorithmically selected sociodemographic, environmental and anthropogenic variables. Intention-to-treat analyses were conducted to estimate the risk reduction of dengue given *Wolbachia* exposure.

**Results:**

Intention-to-treat analyses revealed that, compared with controls, *Wolbachia* releases for 3, 6 and ≥12 months was associated to 47% (95% confidence interval: 25–69%), 44% (33–77%) and 61% (38–78%) protective efficacy against dengue, respectively. When exposed to ≥12 months of *Wolbachia* releases, protective efficacies ranged from 49% (13–72%) to 77% (60–94%) across years. The proportion of virologically confirmed dengue cases was lower overall in the intervention arm. Protective efficacies were found across all years, age and sex subgroups, with higher durations of *Wolbachia* exposure associated to greater risk reductions of dengue.

**Conclusion:**

Results demonstrated that *Wolbachia*-mediated sterility can strengthen dengue control in tropical cities, where dengue burden is the greatest.

## Introduction

Dengue is the most widespread arboviral disease worldwide and has shown sustained increases in burden year on year. The Americas and Southeast Asia routinely account for the majority of global cases.^1^ Vector control remains the primary tool for mitigating the spread of dengue due to the lack of available therapeutics and highly effective vaccines globally. Conventional vector control measures include environmental management, source reduction and insecticide use.^2^^,^^3^ While these measures can reduce the burden of dengue, they are resource intensive and may yield diminishing returns.^3^

*Aedes aegypti* is the primary vector for dengue. Yet, few randomized controlled trials have been conducted for control of vector populations or vector competence to reduce dengue transmission. One trial has used the endpoint of virologically confirmed dengue to examine the impact of introgressing ‘virus-blocking’ strains of *Wolbachia* into field populations of *Ae. aegypti* on dengue incidence in Yogyakarta, Indonesia,^4^ while another trial has used the endpoint of seroconversion to quantify the impact of spatial repellents on human *Aedes*-borne viruses in Iquitos, Peru.^5^

A separate approach employs the use of incompatible insect technique (IIT), which encompasses release of only *Wolbachia*-infected male mosquitoes. Due to cytoplasmic incompatibility (CI),^6^^,^^7^ matings between *Wolbachia*-infected males and uninfected females yield non-viable eggs. Repeated releases of *Wolbachia*-infected males are thus expected to suppress wildtype mosquito populations and reduce disease transmission. IIT avoids disadvantages associated with traditional vector control, including genetic or behavioural resistance to insecticides, off-target effects and the inability to locate cryptic larval sites. IIT further avoids fitness costs arising from exposure to male-sterilizing irradiation, which can reduce mating competitiveness of sterile males in a full sterile insect technique (SIT) programme.^8^ However, imperfect sex-sorting may lead to stable establishment of the released *Wolbachia* strain in the field due to unintentional release of fertile *Wolbachia*-infected female mosquitoes.^9^ While this confers a reduced ability for the *Wolbachia*-established population to transmit dengue (a phenomenon exploited by the Yogyakarta trial above^4^), introgression renders cytoplasmic incompatibility-based IIT ineffective.^9^

To augment vector control in Singapore, we have conducted extensive field trials of *Wolbachia-*mediated IIT targeting *Ae. aegypti*. To reduce the likelihood of stable establishment, we combined IIT with SIT using low-dose irradiation to sterilize residual females during releases of *Wolbachia*-infected males.^10^ As data from randomized trials^11^ are not yet available, observational analyses may be used to ascertain intervention efficacies by adopting a target trial emulation approach, which aimed to use observational data to preserve desirable features of hypothetical randomized trials.^12–14^ This study used a nationally representative test-positive/negative cohort comprising individuals who were tested for dengue via all public hospitals, polyclinics and general practitioners to emulate a cluster-randomized test-negative target trial to ascertain the intervention efficacy of *Wolbachia-*mediated sterility to reduce the incidence of virologically confirmed dengue in Singapore.

## Methods

### Specification of the cluster-randomized test-negative target trial

We specified a cluster-randomized test-negative target trial^12^^,^^13^ to retrospectively evaluate the efficacy of releasing *w*AlbB-infected *Ae. aegypti* male mosquitoes for dengue control via vector population suppression, from epidemiological week (EW) 1 2019 to EW26 2022 in Singapore (Table 1). The emulation approach was used as it could closely mirror important characteristics of actual test-negative cluster-randomized controlled trials, such as (1) constrained randomization, to balance historical disease risk between arms, (2) adjustment for baseline confounders between arms using weighting procedures and (3) the use of test-positive and test-negative comparator groups to avoid selection bias. The target trial was emulated using test-positive/negative databases, which comprised all patients who report to any general practitioner clinic, polyclinic or public/private hospital and were suspect of dengue illness during the trial duration in Singapore (Table 1).

**Table 1:** Summary of the protocol of a target trial estimating differences in dengue transmission risk for individuals residing in *Wolbachia* release sites vs those in control locations who are not

Component	Hypothetical cluster-randomized trial	Target trial emulation
Eligibility	Patients who report for undifferentiated febrile illness to any general practitioner clinic, polyclinic or public/private hospital during trial duration and have home addresses that are in trial locations between EW1 2019 and EW26 2022	Same as hypothetical trial
Intervention strategies	Release of *w*AlB-SG infected male *Ae. aegypti* in home address of participants. An individual is considered directly intervened by *Wolbachia* if home address resides in an area that has sustained releases for more than: (1) 3 months (2) 6 months (3) 12 months	Same as hypothetical trial
Intervention assignment	Townships at historically high risk of dengue transmission pre-selected to either receive intervention or control randomly Townships undergo a constrained randomization strategy for control and intervention to prevent chance imbalance and have same historical dengue incidence rates in the pre-intervention period Townships are the unit of randomization	Four long-term *Wolbachia* field trial townships not randomly pre-selected Control townships undergo a constrained randomization same as the hypothetical trial to match the historical dengue incidence rates of the intervention township in the pre-intervention period Townships are the unit of randomization
Release strategy	Standardized release protocol of 1–7 *w*AlbB-SG males were released per study site resident per week All designated intervention sites would implement intervention at the start of the trial	Standardized release protocol of 1–7 *w*AlbB-SG males were released per study site resident per week Four long-term *Wolbachia* field trial townships either adopted an expanding release approach (release sites were gradually expanded to adjacent neighbourhoods) or targeted release approach (focused releases on areas with high *Aedes aegypti* abundance and persistent dengue transmission)
Blinding	Intervention and participants unblinded to intervention	Same as hypothetical trial
Follow-up	N/A. Repeated tests were removed	Same as hypothetical trial
Enrolment	Unexposed individuals were enrolled if they reported for febrile illness throughout the trial duration and did not reside in an intervention location, whereas exposed individuals were enrolled if they reported for febrile illness and were residing in an intervention location that had interventions for 3, 6 or ≥12 months	Same as hypothetical trial
Primary outcome	Test-positive for dengue using highly sensitive and specific laboratory tests^14^^,^^15^ (internally controlled RT-qPCR assay, dengue non-structural protein 1 (NS1) or IgM)	Same as hypothetical trial
Causal contrast	Intention-to-treat effect	Observational analogue of the intention-to-treat effect
Analysis plan	Intervention (exposure) variable of interest: *w*AlB-SG infected male *Ae. aegypti* at home address will be considered as a binary classification based on time since intervention (see intervention strategies) Outcome variable: test-positive status for dengue at point of testing Doubly robust logistic regression will be used to assess the intervention effect by estimating the aggregate odds ratio, which compares the exposure odds among test-positive cases vs test-negative controls. The null hypothesis is that the odds of residing in the intervention locations are the same among test-positive cases as test-negative controls All analyses will be adjusted for environmental and anthropogenic risk factors associated with dengue transmission risk, and the propensity to receive treatment based on the same risk factors. See detailed statistical analysis plan below	Same as hypothetical trial

### Emulating randomization protocols from cluster-randomized trials for *Wolbachia* interventions

Twenty-six townships in Singapore that were not subject to *Wolbachia* interventions were considered potential locations as control sites. Towns were demarcated planning areas used by government ministries and departments for administrative purposes. While four long-term *Wolbachia* field trial townships were not randomly pre-selected, we emulated constrained randomization protocols for cluster-randomized trials by randomly selecting a set of 12 control townships, such that the historical dengue incidence (2010–2016) of the intervention arm is similar to that of the control arm in the pre-intervention period.^4^^,^^11^^,^^16^ The ratio of 1:3 intervention to controls ensures that a sizeable number of control comparator units were available to generate low levels of bias in the subsequently described statistical inference procedure.^17^ The constrained randomization procedure also helped to prevent chance imbalance in baseline dengue risk due to the small number of intervention (*n* = 4) locations considered (see Supplementary information). All locations practiced the same baseline dengue control protocol in the pre- and post-intervention periods.^2^^,^^3^

### Cohort

Under the Infectious Diseases Act, all laboratory-confirmed cases of dengue are legally mandated for reporting in the national dengue surveillance system. Approval from the Director General of Health, Ministry of Health, was obtained to collect and use the data of dengue-suspected patients, whose blood samples are sent for dengue tests, through a national network of diagnostic laboratories that support private clinics, public polyclinics or public/private hospitals.

This project was exempted from formal bioethics review as it is not considered human biological research, as advised by the Ministry of Health, Singapore. All laboratory tests were performed for clinically directed reasons, and the data from these tests are routinely collected as part of routine dengue surveillance under the Infectious Disease Act, which exempts the need for informed consent.

In Singapore, 133 821 individuals reported for febrile illness and were tested for dengue at the Environmental Health Institute, hospital laboratories and commercial diagnostic laboratories, through general practitioner clinic, polyclinic or public/private hospital from EW1 2019 to EW26 2022. All dengue-suspect patients were tested using either using an internally controlled quantitative reverse transcription polymerase chain reaction (RT-qPCR) assay, dengue non-structural protein 1 (NS1) or immunoglobulin M (IgM) as diagnostic assays to detect dengue virus in serum samples.^3^^,^^11^ We excluded individuals who had more than one residential address in different control or intervention townships and individuals who had been tested at different laboratories with conflicting dengue results. We also excluded individuals who had residential addresses at intervention sites at the time of the test but had not been exposed to *Wolbachia* interventions for at least 3 months, based on exposure criteria described below. For individuals with repeated tests, we classified the individual as test positive if they have a positive test out of multiple tests 4 weeks of each other, otherwise we took the latest test-negative result.

The four intervention townships (Bukit Batok, Choa Chu Kang, Tampines, Yishun) had 7049 tested individuals included in the study period. After constrained randomization, we selected 12 control townships (Bedok, Bishan, Clementi, Geylang, Jurong West, Kallang, Pasir Ris, Punggol, Queenstown, Sengkang, Toa Payoh, Woodlands) with 69 216 tested individuals in the study period. The control arm had an average dengue incidence rate normalized by population size that was <5% different from the intervention arm in the pre-intervention period of EW1 2010 to EW52 2016, indicating good balance in historical dengue risk between arms.

### Outcomes of interest

Analysis considered *Wolbachia* exposure as a binary classification based on the home address of the individual in an intervention sector within an intervention township (*Wolbachia*-exposed) or a control sector within the selected control townships (*Wolbachia*-unexposed). Sectors comprise ≥10 public housing apartment blocks and measured ~0.088 km^2^ on average and are used for planning of surveillance and control for environmental infectious diseases in Singapore.

We subcategorized *Wolbachia* exposure based on whether an individual resides in a sector that experienced sustained *Wolbachia* releases for 3, 6 or ≥12 months due to the time required for releases to induce noticeable vector suppression (see Supplementary information). Home address was defined as the primary place of residence reported on the diagnostic test date. The intervention effect was estimated from an odds ratio comparing the exposure odds (residence in an intervention location for 3, 6 or 12 or more months) among participants who were dengue test-positive versus test-negative controls, with the use of logistic regression (see Statistical analysis below). The null hypothesis was that the odds of residence in an intervention sector would be the same among participants who test positive as that among test-negative controls. Intervention efficacy was calculated as 100 × (1 − odds ratio).

### Characterization of intervention

Male *Wolbachia*-infected *Ae. aegypti* were released twice weekly (weekdays, 0630–1030 hr) at four townships in high-rise public housing estates, to trial whether the intervention could be conducted at large spatial scales and facilitate preparation for larger scale deployment.^18–20^ The locations were also selected as they had characteristics that enabled trials of different release strategies, with Yishun and Tampines being large enough to conduct the expanding release approach and Choa Chu Kang and Bukit Batok having pockets of high mosquito abundance to conduct the targeted release approach (Table 1). Production of male *Wolbachia*-infected *Ae. aegypti* mosquitoes comprised sex separation of male *Aedes* mosquitoes for release, with SIT adopted using low-dose irradiation to sterilize residual females during releases of *Wolbachia*-infected males to reduce the possibility of releasing females (see Supplementary information for full details). The field trial covered 607 872 individuals as of EW26 2022. Bukit Batok, Choa Chu Kang and Yishun towns were subjected to interventions that combined IIT with SIT. Tampines town used the high-fidelity sex-sorting methodology and also progressively adopted SIT protocols to release irradiated mosquitoes from January 2020.^10^^,^^21^ To trial whether *Ae. aegypti* population suppression could be sustained over increasingly larger areas, an expanding release strategy was adopted in two large towns (Yishun, Tampines), where release sites were gradually expanded to adjacent neighbourhoods. In Bukit Batok and Choa Chu Kang towns, a targeted release approach was adopted, which focused releases on areas with high *Ae. aegypti* abundance and persistent dengue transmission (Table 2, see Supplementary information for full details). Adult *Ae. aegypti* populations in release and control sites were monitored using Gravitraps, with an average of six Gravitraps deployed per apartment block.^22^

**Table 2:** Summary of *Wolbachia* intervention approaches over 4 townships

Township	Bukit Batok	Choa Chu Kang	Tampines	Yishun
Intervention start date	EW23 2020	EW20 2020	EW39 2018	EW27 2018
Study end date	EW26 2022	EW26 2022	EW26 2022	EW26 2022
Intervention time (weeks)	109	112	197	209
Total township size (m^2^)^&^	627 441	1 145 559	5 088 046	3 473 690
Production approach^**^	IIT-SIT	IIT-SIT	High-fidelity sex-sorting^&&^	IIT-SIT
Frequency of release	Twice weekly	Twice weekly	Twice weekly	Twice weekly
Release strategy^***^	Targeted^#^	Targeted	Expanding^##^	Expanding
Number of mosquitoes released	1–7 *w*AlbB-SG males were released per study site resident per week			
Total number of mosquitoes released (rounded to thousands)	17 139 000	14 598 000	109 432 000	77 659 000
Township population covered by release over study period	40 132	64 672	272 048	231 020

& Total area of public housing estates subject to release in respective townships in EW26 2022

^*^Computed as (sum of area of releases multiplied by weeks of release until end of study period) over (total area of township multiplied by total release weeks). Areas were considered covered once they receive at least 6 months of *Wolbachia* interventions

^**^The IIT-SIT approach and high-fidelity sex-sorting are detailed in Supplementary information section 1 and has been previously characterized^10^^,^^13^

^***^Denotes approach to releasing *Wolbachia*-infected males

# Targeted approach that focused releases on areas with high *Aedes aegypti* abundance and persistent dengue transmission

## Expanding (‘rolling’) approach where release sites were gradually expanded to adjacent neighbourhoods

&& IIT-SIT increasingly adopted into production protocol from August 2020 onwards

### Covariates

We extracted a comprehensive set of spatially explicit variables to characterize environmental heterogeneity across sectors. Covariates considered prior to variable selection include (1) vegetation maps with areas classified across multiple vegetation types including forest and managed vegetation to signify availability of natural breeding sites and nectar availability for male mosquitoes. (2) The averaged Normalized Difference Vegetation Index per sector, as an alternative measure of vegetation. (3) To represent host density and urban breeding habitat availability, data on the locations of all public housing estates where >75% of Singapore’s resident population reside were obtained. Utilizing residential location and resale data, the average age of public housing residences was collected as older age is a well-established risk factor for higher mosquito abundance.^23^ (4) Average residence price over the study duration as a proxy for household income and socioeconomic status. (5) Building height was calculated according to the number of floors with an average height of 3 m. (6) The number of condominiums/landed properties was collected within each sector representing additional hosts being available. The percentage cover of built area was calculated as a sum of all residential, commercial and industrial buildings, representing the level of urbanicity, which has been associated with *Ae. aegypti* presence.^14^ (7) The major open drainage network for Singapore was obtained from the Public Utilities Board and has been previously shown as a key breeding site for mosquitoes around public housing apartments.^24^ The average distance of each block within a sector to a drain was measured as well as the length of the network within the sector. (8) Well-established meteorological variables that are known to affect mosquito survival or fecundity were collected. These included daily mean, maximum and minimum temperature, total rainfall and wind speed, which were obtained from 21 local weather stations. Hourly dewpoint and ambient ground air temperature were also taken from remote sensing measurements to estimate relative humidity over the time period using standard formula. These values were aggregated at a weekly level to correspond with the temporal frequency of dengue test-positive/negative data. Data sources and processing procedures are explicitly detailed in the Supplementary information.

### Statistical analysis

Baseline characteristics of the cohort were presented as mean and standard deviation or as frequency and percentage. Standardized mean differences (SMDs) were used to evaluate balance between intervention and non-intervention arms, with SMD < 0.1 indicating good balance.

To estimate the effect of *Wolbachia* interventions on the risk of dengue, we employed a doubly robust logistic regression framework, which weighs samples based on inverse probability weights estimated using propensity score models and then further adjusts for covariates used to learn propensity scores in the outcome regression. First, to estimate propensity score models, we used the considered set of covariates described above as the independent variables and *Wolbachia* exposure for at least 3, 6 and 12 months as separate outcomes of interest. Prespecified variables included age and sex, with other factors selected using high-dimensional regression and additional trimming of highly multicollinear covariates (see Supplementary information). Sensitivity analyses later also indicated that variable selection procedures did not influence *Wolbachia* protective effect estimates.

We adjusted for differences in baseline characteristics and the propensity to be selected as a treatment site between intervention/non-intervention arms through inverse probability weighting, incorporating the selected covariates. A propensity score of belonging to the intervention arm was computed using a logistic regression that used the abovementioned covariates as explanatory terms. Inverse probability weights were computed as 1/propensity score for tested individuals who were *Wolbachia* exposed and 1/(1 − propensity score) for tested individuals who were not *Wolbachia* exposed.^25^ SMDs were used to assess covariate balance after inverse probability weighting. Thereafter, odds ratios (ORs) of being dengue test positive between the intervention and control groups were estimated using a logistic regression model, with inverse probability weights applied. A doubly robust approach was employed for this model, where covariates used to construct inverse probability weights were included in each model specification as explanatory variables. This approach was used as it ensured robustness against model misspecification in either the propensity score models or in the outcome regression.

To account for within-town dependencies, we relied on cluster bootstrap based on 1000 clustered resamples. Balanced bootstrap resampling based on town membership can account for within-town dependencies and has been used as a competitive approach to analyse hierarchical data.^17^^,^^26^ The associated bootstrap percentile-based confidence interval was used to construct the 95% confidence interval for ORs, and findings were considered to be statistically significant when the 95% confidence intervals for ORs did not cross 1.

### Subgroup analysis

We repeated all analysis by subsetting to intervention townships and specific years (2019, 2020, 2021, 2022) to examine any potential differences in intervention effect by location, and between epidemic and inter-epidemic years. We also repeat analysis by age (<20, 20–65, 65+) and sex (male, female) subgroups as dengue risk may be mediated by immunity levels in each age group or sex. Here, we conducted subgroup analyses by re-estimating ORs using the aforementioned statistical procedures, but only using individuals within that specific subgroup.

### Robustness checks

We conducted a battery of sensitivity analyses to ensure the robustness of our model estimates. We (1) repeated all analysis without adjustment for covariates in the main logistic regression step; (2) re-randomized our allocation of controls 1000 times and repeated our analysis by using the newly allocated controls arm, and compared our primary estimate of intervention efficacy against the empirical distribution of re-randomized intervention efficacies; (3) used the full set of covariates, instead of the pre-selected covariates in our main analysis, to recompute ORs and intervention efficacies; (4) conducted in-space placebo checks on control sites, taking each allocated control site as the allocated placebo-intervention site and re-estimated ORs and intervention efficacies by comparing test-negative and test-positive individuals in the placebo-intervention vs other control sites; (5) conducted placebo checks on intervention sites, taking each intervention site in 52 and 104 weeks before the actual intervention as the intervention period, and recomputed intervention efficacies among allocated controls and interventions based on this placebo-intervention period; (6) recomputed intervention efficacies using cluster bootstrap on the sector rather than town resolution; (7) recomputed intervention efficacies using logistic regression without weighting, with algorithmically selected covariates, full set of covariates and no covariates in the outcome regression set; (8) recomputed intervention efficacies using two separate cluster-level summary measures^27^^,^^28^ and (9) recomputed intervention efficacies using an alternate propensity score matching procedure, where exact matching of calendar times was done for both *Wolbachia*-exposed/unexposed individuals to control for differences in temporal risk, and nearest-neighbour matching for all other environmental/anthropogenic covariates.

All analysis was conducted in R.4.3.1.

## Results

### Suppression of *A. aegypti* populations in field trial sites

**Figure 1:**
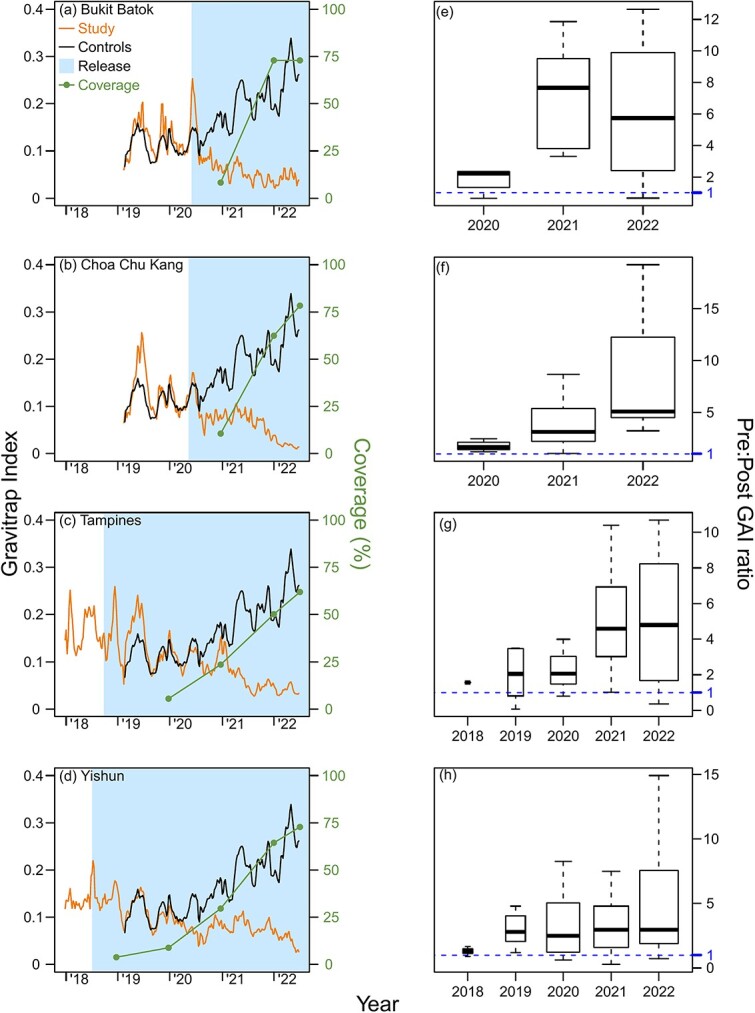
Weekly average, town-level Gravitrap *Aedes aegypti* index (GAI) and *Wolbachia* coverage (%) from 2018 to 2022 in the intervention sites of (a) Bukit Batok, (b) Choa Chu Kang, (c) Tampines and (d) Yishun. The average GAI from the 12 controls is plotted for comparison. GAI is defined as the mean number of female adult *Ae. aegypti* caught per functional Gravitrap per week, hence proxies for adult *Ae. aegypti* abundance in public housing areas in release area of each town. The geographical coverage (%) represents the percentage of areas within the town that is covered by *Wolbachia* interventions for at least 6 months and is calculated at the end of each year. Points represent coverage of *Wolbachia* interventions by the end of each year. The 6-month mark for coverage is based on the time it takes *Wolbachia* release to have ~80% suppressive efficacy on *Ae. aegypti* abundance. The corresponding ratios of pre- and post-intervention GAIs at the same township is also plotted in (e), (f), (g) and (h), per year, on a per-township basis. The threshold of 1 indicates no difference between pre- and post-GAIs for a specific sector in that specific year vs the pre-intervention period, and values >1 indicate lower GAIs in the post-intervention period

Suppression of adult wildtype *Ae. aegypti* populations was demonstrated across the four field trial sites, with the Gravitrap *A. aegypti* index (GAI) reduced as *Wolbachia* coverage increased across each township. When > 50% coverage was achieved by EW1 2022, the town-level GAI plunged >0.05 for all sites (Fig. 1, Supplementary information).

### Study characteristics

Among 133 821 individuals who reported for febrile illness from EW1 2019 to EW26 2022, in the intervention and control arms, 76 265 (56.99%) were included in the study. Baseline demographic and spatial–temporal characteristics between *Wolbachia*-exposed and unexposed groups before and after inverse probability weighting are presented in Table 3. Characteristics were well matched after inverse probability weighting (Table 3) with small differences in baseline characteristics between both groups.

**Table 3:** Baseline characteristics of study population pre- and post-*Wolbachia* releases in intervention and pre-selected control group, at the sector resolution (the numbers in bracket represent standard deviation for each characteristic)

	Intervention		Control		
	Observed	Weighted	Observed	Weighted	SMD^**^
Pre-intervention (EW1 2010 to EW52 2016)					
Pre-intervention dengue incidence per 100 000^*^	112.22 (116.53)		113.78 (110.34)		
Post-intervention (EW1 2019 to EW26 2022)					
Post-intervention dengue incidence per 100 000^*^	158.8 (93.19)		294 (230.72)		
Dengue test positive (%)^#^	13.6 (0.004)	13.2 (0.001)	21.7 (0.002)	21.2 (0.002)	
Covariates					
Male (%)	50.19 (0.006)	49.49 (0.002)	51.09 (0.002)	51.09 (0.002)	0.022
Age (years)	49.65 (23.8)	45.09 (24.65)	45.25 (23.68)	45.42 (23.69)	−0.02
NDVI (Vegetation Index)	0.33 (0.05)	0.32 (0.05)	0.33 (0.05)	0.33 (0.05)	−0.08
Area within 300 m of a waterbody (%)	0.18 (0.25)	0.29 (0.29)	0.37 (0.41)	0.36 (0.4)	−0.1
Public housing height (m)	31.52 (4.66)	33.99 (4.45)	37.88 (10.21)	37.53 (10.19)	−0.28
Public housing age (years)	32.33 (7.96)	28.56 (11.16)	29.07 (11.07)	29.23 (11)	−0.07
Number of public housing units	722.4 (877.74)	1080.62 (1180.89)	713.71 (711.19)	714.96 (709.31)	0.28
Distance of centroid to drainage network (m)	359.87 (245.88)	354.52 (236.27)	448.77 (322.88)	446.37 (322.08)	−0.23
Length of drainage network (m)	88.08 (190.24)	48.04 (133.81)	43.52 (117.89)	44.61 (119.15)	−0.02
Forest area (%)	0 (0)	0 (0)	0.0004 (0.004)	0.0004 (0.004)	−0.09
Grass area (%)	0.001 (0.006)	0.002 (0.008)	0.008 (0.03)	0.008 (0.03)	−0.18
Total vegetation area (%)	0.02 (0.04)	0.03 (0.05)	0.03 (0.06)	0.03 (0.06)	0.13
Building area (%)	0.26 (0.04)	0.25 (0.04)	0.25 (0.06)	0.25 (0.06)	−0.04
Maximum temperature (°C)^1^	31.87 (1.05)	31.94 (0.99)	31.93 (1.06)	31.98 (1.01)	−0.01
Mean temperature (°C)	27.86 (0.84)	27.90 (0.76)	28.10 (0.84)	28.14 (0.80)	−0.2
Rainfall (mm)	7.36 (5.47)	6.55 (5.30)	6.37 (5.24)	6.38 (5.21)	0.02
Mean wind speed	8.18 (2.14)	9.11 (2.44)	9.28 (2.67)	9.18 (2.61)	0.01
Relative humidity	79.87 (2.83)	79.69 (3.24)	79.52 (3.28)	79.45 (2.87)	0.04

^*^Pre-intervention period dengue incidence denotes number of dengue cases per 100 000 per sector annually

# Post-intervention percentage of dengue test-positives compared to total number of tests per sector. Only data on dengue tests were available 2016 onwards

^1^Maximum temperature was calculated by taking maximum of temperature across all sectors within intervention or control groups. Length of drainage network and number of public housing units were calculated by taking sum across all sectors within intervention or control groups. The remaining characteristics were calculated by averaging across all sectors within intervention or control groups. All the calculations were done for the specified time period

^**^Standardized mean differences (SMDs) after inverse probability weighting of intervention (*Wolbachia*-exposed) and controls (*Wolbachia*-unexposed) individuals. Tested individuals were considered *Wolbachia*-exposed here if they reside in a place of residence that has sustained *Wolbachia* interventions for ≥3 months

### Efficacy of *Wolbachia* releases in reducing risk of dengue

Among dengue-tested individuals residing in areas that were *Wolbachia* exposed for at least 3 months, the percentage of individuals who tested positive for dengue (13.6%, 956 of 7049 individuals) was lower compared to the *Wolbachia*-unexposed (21.7%, 14 986 of 69 216 individuals) group.

In primary analysis, *Wolbachia* exposure for at least 3, 6 or 12 months was associated to a lower risk of being test positive for dengue. Higher periods of exposure associated to greater levels of protective efficacy—at 47% (OR: 0.53 [0.31–0.75]), 44% (OR: 0.56 [0.33–0.76]) and 61% (OR: 0.39 [0.22–0.62]) for at least 3, 6 and 12 months of *Wolbachia* exposure, respectively. Protective efficacies were higher in epidemic years (2019, 2020, 2022) vs inter-epidemic years (Table 4).

**Table 4:** Odds ratios (ORs) and test-positive percentages for different *Wolbachia* exposure categories and across year, age and sex subgroups

	OR	Test positive(%)^##^		OR	Test positive (%)		OR	Test positive (%)		OR	Test positive (%)		OR	Test positive (%)	
Exposure time^#^	(95% CI)*	Exposed	Unexposed	(95% CI)	Exposed	Unexposed	(95% CI)	Exposed	Unexposed	(95% CI)	Exposed	Unexposed	(95% CI)	Exposed	Unexposed
By year	2019			2020			2021			2022			Aggregate		
3 months +	**0.51** * (0.23–0.85)	7.7% (37/482)	17.2% (3839/22 377)	**0.59** * (0.28–0.83)	21.9% (525/2395)	27.5% (6549/23 852)	0.73 (0.27–1.88)	6.4% (147/2294)	9.5% (1121/11 787)	**0.37** * **(0.2–0.58)**	13.2% (247/1878)	31.0% (3477/11 200)	**0.53** * **(0.31–0.75)**	13.6% (956/7050)	21.7% (14 986/69 216)
6 months +	**0.49** * **(0.13–0.74)**	6.1% (19/314)	17.2% (3839/22 377)	**0.58** * **(0.31–0.83)**	22.4% (376/1679)	27.5% (6549/23 852)	0.59 (0.36–1.09)	6.3% (137/2191)	9.5% (1121/11 787)	**0.41** * **(0.24–0.65)**	13.4% (235/1751)	31.0% (3477/11 200)	**0.56** * **(0.33–0.76)**	12.9% (767/5935)	21.7% (14 986/69 216)
12 months +	**0.23** * **(0.06–0.4)**	3.2% (3/94)	17.2% (3839/22 377)	**0.48** * **(0.36–0.58)**	15.9% (98/617)	27.5% (6549/23 852)	**0.51** * **(0.28–0.87)**	6.2% (108/1737)	9.5% (1121/11 787)	**0.4** * **(0.23–0.63)**	13.4% (219/1635)	31.0% (3477/11 200)	**0.39** * **(0.22–0.62)**	10.5% (428/4068)	21.7% (14 986/69 216)
By age and gender	**0–20**			**20–65**			**65+**			**Male**			**Female**		
3 months +	0.59 (0.24–1.01)	12.4% (93/750)	18.4% (1881/8549)	**0.54** * **(0.32–0.79)**	16.5% (664/3462)	24.8% (10 427/31 612)	0.54 (0.33–1.01)	8.7% (195/2237)	15.5% (2584/16 637)	0.67 (0.29–1.04)	14.6% (498/3404)	22.1% (7236/32 686)	**0.39** * **(0.31–0.53)**	11.1% (374/3376)	19.6% (6167/31 389)
6 months +	0.75 (0.22–1.16)	12.7% (80/630)	18.4% (1881/8549)	**0.54** * **(0.36–0.7)**	15.6% (525/3367)	24.8% (10 427/31 612)	**0.53** * **(0.26–0.82)**	8.3% (158/1912)	15.5% (2584/16 637)	**0.67** * **(0.29–1)**	13.7% (393/2865)	22.1% (7236/32 686)	**0.45** * **(0.35–0.56)**	10.9% (309/2846)	19.6% (6167/31 389)
12 months +	**0.34** * **(0.13–0.62)**	8.9% (38/415)	18.4% (1881/8549)	**0.38** * **(0.24–0.55)**	12.5% (283/2271)	24.8% (10 427/31 612)	0.46 (0.22–1.21)	7.7% (105/1358)	15.5% (2584/16 637)	**0.49** * **(0.25–0.84)**	11.9% (238/1999)	22.1% (7236/32 686)	**0.27** * **(0.16–0.41)**	8.3% (161/1943)	19.6% (6167/31 389)

^*^Denotes an OR that is less than 1 with 95% confidence intervals (CIs) that do not contain 1. These ORs show that there a significant protective effect of *Wolbachia* interventions on the risk of dengue. Figures are also bolded to denote statistical significance for clarity. ORs are estimated using doubly robust logistic regression with weights for each individual estimated using inverse probability weighting. Cluster bootstrap at the town resolution was used to obtain CIs to account for town-specific spatial clustering of data and the intervention

^**^Unexposed group taken as the pre-randomized set of 12 controls

# An individual testing for febrile illness is considered *Wolbachia*-exposed if the individual resides in a sector with 3, 6 or ≥12 months of sustained *Wolbachia* release

## Unweighted percentages of individuals testing positive in *Wolbachia*-exposed and *Wolbachia*-unexposed sectors

By stratifying analysis across years, age and sex subgroups and taking *Wolbachia* exposure at ≥12 months as the reference, the highest level of protective efficacy was in the adolescent (66% protective efficacy, OR: 0.34 [0.13–0.62]) and adult age groups (62% protective efficacy, OR: 0.38 [0.24–0.55]) when compared across age groups and in females (73% protective efficacy, OR: 0.27 [0.16–0.41]) when compared against both sexes. Protective efficacies were found across all subgroups, with longer durations of exposure similarly associated to greater risk reductions of dengue—supporting consistent biologic replication of the intervention (Table 4).

### Sensitivity analyses

We conducted a battery of sensitivity analyses to ensure the robustness of our model estimates. Repeating all analysis without adjustment for covariates, or inclusion of all available covariates in main logistic regression step did not change intervention efficacy estimates. Repeating our analysis on unweighted logistic regressions using the algorithmically selected covariates without adjustment for covariates, or inclusion of all available covariates in the outcome regression step did not change intervention efficacy estimates. Use of cluster-level summary measures to compute intervention efficacy reproduced significant intervention efficacies. Re-randomizing our allocation of controls into the control arm 1000 times and repeating our analysis by using newly allocated controls arms did not change intervention efficacy estimates vs our primary estimate of intervention efficacy. We further did in-space placebo checks on control sites, taking each allocated control site as the allocated placebo-intervention site and re-estimated protective efficacies by comparing test-negative and test-positive individuals in the placebo-intervention vs other control sites. There were few significant/positive intervention efficacies demonstrated in the placebo-control sites with the grand mean of OR estimates centred closely to 1. Lastly, we conducted in-time placebo checks on intervention sites, where we took placebo interventions in the actual intervention site before the intervention began and demonstrated that there were no significant/positive protective effects in the intervention sites in the placebo-intervention period. Re-running our cluster bootstrap inference on the sector, rather than town level, also demonstrated that we could recover the intervention efficacy point estimates, albeit with narrower confidence intervals due to the finer spatial resolution used for bootstrapping. Explicit results on all sensitivity analyses are provided in the Supplementary information.

## Discussion

Releases of *w*AlbB-infected *Ae. aegypti* male mosquitoes reduced the incidence of dengue by 61% in areas that received ≥12 months of sustained intervention (Table 4). Across all intervention townships, inter-epidemic and epidemic years, age and sex subgroups, the proportion of individuals testing positive for dengue was lower vs the control arm, demonstrating consistent biological replication of the intervention effect (Table 4).

Variation across years was likely due to different dengue incidence rates at control sites, which are influenced by the national situation. Outbreak years (2019, 2020, 2022) typically yield higher efficacy than non-outbreak years (2021). The reduced efficacy following 2019 was also likely due to the expansion to Choa Chu Kang, which had lower efficacy, in the later years.

We emulated a cluster-randomized test-negative controlled target trial in this study, which has been employed to study the epidemiological efficacy of field interventions, such as *Wolbachia.*^4^^,^^11^^,^^16^ By employing a large and representative cohort of patients who have suspect dengue illness and were tested for dengue, across all major diagnostic laboratories through public hospitals, general practitioner clinics and polyclinics, we were able to emulate a cluster-randomized controlled target trial and incorporate key study characteristics, such as (1) constrained randomization, which enabled intervention and control arms to have balanced dengue risk in the pre-intervention period; (2) the use of test-positive and test-negative comparator groups, to avoid selection bias at the point of testing and enable detection of virologically confirmed dengue cases and thereby (3) enabling causal identification of the protective efficacy of *Wolbachia* on dengue.

The protective efficacy of *w*AlbB-infected *Ae. aegypti* male mosquito releases are consistent with previous laboratory and entomological field observations. Release of incompatible *Ae. aegypti* male mosquitoes can drive profound suppression of wildtype *Ae. aegypti* mosquitoes.^9^^,^^21^^,^^29–31^ While previous field trials have demonstrated the protective efficacy of *w*Mel introgression in reducing dengue risk,^4^ and the efficacy of incompatible insect technique to reduce town-level dengue incidence,^32^ no study has yet examined the effect of incompatible insect technique in reducing individuals’ risk of dengue. Our study combined data from a large-scale field trial of *w*AlbB-infected *Ae. aegypti* male mosquito releases, nationally representative dengue testing databases, a large set of potential environmental and anthropogenic confounders and utilized a robust cluster-randomized trial emulation framework to demonstrate the protective efficacy of the technology on dengue.

The technology has several key advantages. (1) While we have only demonstrated the protective efficacy of the approach for dengue, as it is the only *Aedes*-borne disease in constant circulation in the study setting, this efficacy should be similar against other diseases transmitted by *Ae. aegypti*, such as Zika, chikungunya and yellow fever, as the strategy aims to suppress vector populations. (2) Baseline studies demonstrate the high public acceptance towards the intervention,^33^ which involves releases of non-biting males only. (3) In earlier studies, 100% CI is induced by *w*AlbB in *w*AlB-SG—the *Wolbachia* strain that was used in Singapore’s field trials.^10^ Another study also demonstrated a long-term stability of the *w*AlbB–*Ae. aegypti* association, with neither alteration of CI expression nor attenuation of the *Wolbachia* titre.^34^ (4) Lastly, while dengue virus may evolve resistance to *Wolbachia* under the *Wolbachia*-introgression approach,^35^ our approach suffers no drawbacks related to *Wolbachia*-associated selective pressure of viruses.

Compared to the alternative *Wolbachia-*introgression approach, release of male *Wolbachia-*infected mosquitoes to suppress the wildtype *Ae. aegypti* population has a higher upfront implementation cost due the need for higher-volume releases compared to introgression (at least at the outset of the intervention when wildtype numbers are high), the need for continual releases to sustain the reductions in *Aedes* abundance, as well as the need for sex separation and irradiation of produced mosquitoes. However, longer term risks and costs should also be considered; e.g. in the *Wolbachia-*introgression approach, the possibility of dengue virus evolving resistance to *Wolbachia*^36^ and the potential impact of climate change (increased temperatures) on *Wolbachia* stability^37^ may add to programme costs. In our study setting, implementation of a *Wolbachia* suppression programme at a national level has been demonstrated to be cost-effective at an intervention efficacy threshold of 40%, which has been met in this study.^38^

The test-negative design also relies on several key assumptions that,^13^ while limiting the applicability of the design, have been met in our study. These include the requirements that (1) other febrile illnesses were not associated to the intervention, (2) highly sensitive and specific tests were used to determine test-positive/negative status, (3) uncensored sampling of controls, (4) both test-positive/negative groups report for illness throughout the test duration, (5) the efficacy of the intervention not being associated with health care–seeking behaviour, and (6) relative propensity of treated and untreated populations to seek health care is nondifferential among cases and controls. These assumptions were met in our study. Firstly, *Wolbachia* interventions were highly specific in targeting dengue within the study setting. Secondly, only highly sensitive and specific tests were used (RT-qPCR assay, NS1 or IgM) to differentiate between test-positives and negatives. Thirdly, nonzero counts of test-positives and negatives have been recorded for the study period for all epidemiological weeks. Fourthly, dengue is legally notifiable in the study setting, with Singapore having an extensive dengue testing and surveillance system and database—which this study relied on. Furthermore, by augmenting the statistical analysis plan with propensity scoring procedures, we also balanced key anthropogenic, environmental and socioeconomic confounders such as house prices and building age between intervention and control arms. This helped to minimize the risk of differential propensity to seek health care between arms if said behaviour was correlated to the confounders in the post-intervention period. However, while a large battery of environmental and anthropogenic characteristics that may potentially confound dengue risk have been adjusted for in the analysis, there may be residual or unobserved confounding of intervention efficacy estimates. By further employing comprehensive in-space and in-time placebo tests, we also demonstrated that for all generated allocations of intervention/control arms under the constrained randomization procedure and in the pre-intervention period, estimated placebo-intervention efficacies were centred around null. This further demonstrated that our analytical plan was not affected by the risk of differential health care–seeking behaviour in the pre-intervention period between the allocated intervention and control arms, or between placebo-intervened and controls. Unlike an idealized randomized controlled trial that enrols exposed and unexposed individuals at similar calendar times, the staggered adoption setting of the field trials mean that exposed and unexposed individuals are enrolled at varying time points, which may lead to biases if there were temporal variations in dengue incidence. Our study time, however, comprises both inter-epidemic and epidemic seasons of dengue transmission (2019–2022), and these variations are likely to be averaged out across the long timeframe. Subgroup analysis by year was also conducted to examine year-on-year effects and remove temporal variations from intervention efficacy estimates. Lastly, it was difficult to directly examine the relationship between protective efficacies and *Aedes* abundance as proxied by the Gravitrap index in our study, as full Gravitrap coverage in the study setting has only been accomplished in 2020.

Release of *Wolbachia*-infected male *Ae. aegypti* is a novel method for the control of dengue. It should, however, not be viewed as a replacement for baseline vector control methods. The technology can complement conventional approaches, such as source reduction and community engagement, in further reducing dengue transmission. In our experience, the protective efficacy of the intervention on dengue, as well as its entomological efficacy are likely to be maximized if it is used to complement and enhance conventional vector control measures, rather than replace them.

## Supplementary Material

SI_18072024_taae103

## Data Availability

The databases used in this study are the property of the Ministry of Health, Singapore, and were shared under the Infectious Disease Act. Permission to access dengue case data should be obtained from the Ministry of Health, Singapore. Request for permission of use should be directed to Diyar_Mailepessov@nea.gov.sg.

## Code availability statement

All code used to reproduce the results of this study accompany the submission.
